# Novel HIV-1 Knockdown Targets Identified by an Enriched Kinases/Phosphatases shRNA Library Using a Long-Term Iterative Screen in Jurkat T-Cells

**DOI:** 10.1371/journal.pone.0009276

**Published:** 2010-02-17

**Authors:** Sylvie Rato, Sara Maia, Paula M. Brito, Leonor Resende, Carina F. Pereira, Catarina Moita, Rui P. Freitas, José Moniz-Pereira, Nir Hacohen, Luis Ferreira Moita, Joao Goncalves

**Affiliations:** 1 URIA-Centro de Patogénese Molecular, Faculdade de Farmácia, Universidade de Lisboa, Lisboa, Portugal; 2 Instituto de Medicina Molecular, Faculdade de Medicina da Universidade de Lisboa, Lisboa, Portugal; 3 Division of Rheumatology, Allergy and Immunology, Center for Immunology and Inflammatory Diseases, Massachusetts General Hospital, Charlestown, Massachusetts, United States of America; 4 Broad Institute of Harvard and Massachusetts Institute of Technology (MIT), Cambridge, Massachusetts, United States of America; Institut Pasteur, France

## Abstract

HIV-1 is a complex retrovirus that uses host machinery to promote its replication. Understanding cellular proteins involved in the multistep process of HIV-1 infection may result in the discovery of more adapted and effective therapeutic targets. Kinases and phosphatases are a druggable class of proteins critically involved in regulation of signal pathways of eukaryotic cells. Here, we focused on the discovery of kinases and phosphatases that are essential for HIV-1 replication but dispensable for cell viability. We performed an iterative screen in Jurkat T-cells with a short-hairpin-RNA (shRNA) library highly enriched for human kinases and phosphatases. We identified 14 new proteins essential for HIV-1 replication that do not affect cell viability. These proteins are described to be involved in MAPK, JNK and ERK pathways, vesicular traffic and DNA repair. Moreover, we show that the proteins under study are important in an early step of HIV-1 infection before viral integration, whereas some of them affect viral transcription/translation. This study brings new insights for the complex interplay of HIV-1/host cell and opens new possibilities for antiviral strategies.

## Introduction

Despite all the efforts in the last three decades for the development of new drugs for acquired immune deficiency syndrome (AIDS) treatment, human immunodeficiency virus type 1 (HIV-1)/AIDS continue to be one of the major human health setbacks of our days [1]. HIV therapies (HAART) developed so far (reviewed in [2]) although powerful, effective against HIV and capable of prolonging life and health of the infected individuals, are still not able to cure AIDS [3]. The ability of HIV to establish latent reservoirs early on the course of infection and its capacity to mutate at a high rate, leading to the emergency of resistant viruses, are the major concerns for the current therapies [4]. Therefore, it is crucial to identify novel drug targets and new therapeutic strategies to combat AIDS. A better understanding of the virus and host-cell interplay could hopefully provide valuable insights into the molecular interactions involved in various steps of retroviral replication. The knowledge of these novel critical players can lead to the development of more adapted and effective therapeutic approaches for eradication of HIV-1 infection [5].

During the past years, several studies have been focused in the identification of host factors that assist HIV-1 during the different steps of its replication cycle [5], [6]. Nevertheless, due to the complexity of the interaction between the virus and the host cell, numerous proteins and mechanisms are yet to be discovered. Recently, different studies using genome-wide RNA interference (RNAi) screens were performed to discover new cellular proteins important for HIV-1 replication [7]–[10]. Three of these screens used siRNA libraries and were transiently expressed in HeLa or HEK293T cells [7]–[9]. Recently, Kuan-Teh Jeang and co-workers performed a loss of function screen with short-hairpin-RNA (shRNAs) cloned in lentiviral vectors to allow the constitutive expression of the shRNAs in Jurkat T-cells [10]. All these screens are based in RNAi libraries that cover all human genes. Nonetheless, despite using similar strategies, the degree of functional overlap between the identified proteins in the different screens was very low. Importantly, these studies brought noteworthy knowledge on HIV-1/host interaction by identifying many cellular proteins that had not yet been related to HIV-1 infection. Moreover, the diversity of identified proteins suggests a vast complexity of host-virus interplay.

Differently than previous studies, in this work we used a smaller library enriched for human kinases and phosphatases, narrowing down the heterogeneity and possible off-target genes that could result from a genome-wide RNAi screen. Similarly to the work of Kuan-Teh Jeang and co-workers, we used Jurkat cells to access specific T-cell genes important for HIV-1 replication but with the additional goal of identifying cellular drug targets for an antiviral strategy. Furthermore, in contrast to the previous study, selection of HIV-1 resistant cells was dependent on viral expression (direct readout) instead of cell death due to viral infection (indirect readout). These differences are expected to complement and improve the goal of discovering novel HIV-1 knockdown targets.

Amongst all proteins considered, kinases and phosphatases are probably the most important regulators of biological and signal pathways. These proteins are key complementary players in protein phosphorylation, a well-characterized biochemical process for reversible regulation of protein activity [11]. Moreover, since kinases and phosphatases are enzymes whose catalytic activity can be effectively and specifically turned off by active site-directed inhibitors, they constitute nowadays the largest subset of the druggable genome, the so-called “kinome” and “phosphatome”. Thus, we can envision that kinase/phosphatase modulation is a promising approach for the development of novel therapeutic strategies to overcome antiviral drug resistance [12], [13]. In this context, the study of kinase and phosphatases genes and their function during HIV-1 replication may not only contribute to a better knowledge of HIV-cell interaction but also may lead to the discovery of new cellular targets for HIV-1 therapy.

With this iterative shRNA screen in Jurkat cells we identified 14 different cellular proteins, involved in several cellular pathways that are essential for HIV-1 replication. Furthermore, our results indicate that the majority of these proteins are not involved in viral integration, being important during entry into the cell and/or uncoating and also affecting viral transcription/translation.

Our results bring not only new insights to the complexity of the HIV-1/host interaction but also open possibilities of exploring novel therapeutic strategies for the treatment of AIDS by targeting kinases and phosphatases.

## Materials and Methods

### Cell Lines and Culture Conditions

Jurkat E6-1 T-cells obtained through the NIH AIDS Research and Reference Reagent Program (MD, USA, contributor Dr. Arthur Weiss) were cultured in RPMI-1640, supplemented with 10% FBS (RPMI-10). HEK293T (ATCC, VA, USA) and HeLa-P4 cells (HeLa-CD4-LTR-β-gal, AIDS Reagent, MD, USA, contributor Dr. Richard Axel) were cultured in DMEM supplemented with 10% FBS (DMEM-10). Jurkat cells expressing shRNA (shRNA clones) were cultured in RPMI-10 supplemented with 2 µg/ml of puromycin (Sigma, MO, USA). All cell cultures were maintained at 37°C in 5% CO_2_. All cell culture media and reagents, otherwise indicated, were from Lonza (Basel, Switzerland).

### Viral Production

HEK293T cells were transfected, by calcium phosphate method, with pNL4-3-r-HSAS (AIDS reagent, contributors Drs. Beth Jamieson and Jerome Zack) or pHIV-1_NL4-3_ plasmids (AIDS reagent, contributor Dr. Malcolm Martin) to produce HIV-HSA or HIV-1_NL4-3_ virions, respectively. After 48 h, virions were collected from supernatant cultures, measured by p24^CA^ ELISA (AIDS & Cancer Research Program, NCI Frederick, MD, USA) and used to infect Jurkat cells.

### Infection Assays

Jurkat cells, shRNA library and individual cell clones were infected with HIV-HSA or HIV-1_NL4-3_ at the indicated Multiplicity of Infection (MOI). For this purpose, cells were ressuspended in a viral preparation and subject to spinoculation [14]. After 6 h, cells were washed in PBS (1×) and medium was replaced. During the 7 day-infection assay, medium was replaced at day 4. HIV-1 replication was monitored in all experiments by p24^CA^ ELISA (AIDS & Cancer Research Program).

### Lentiviral shRNA Library Screen

Lentiviral shRNA library composed by 3–5 shRNA for each gene was enriched for human kinases and phosphatases by rearraying LKO.1 shRNA constructs obtained from the RNAi Consortium (TRC) (Broad Institute, MA, USA). High-titer lentiviral production was obtained after transfection of HEK293T cells with the shRNA-encoded library. The plasmids included in the lentiviral packaging mix encode the key structural viral packaging genes and a heterologous viral envelope gene in a three-plasmid lentivirus packaging system [15]. We collected the cell supernatant containing a highly infectious pool of VSV-G pseudotyped shRNA-encoding lentiviral particles and used it for transduction of Jurkat cells.

Jurkat cells were transduced with the lentiviral shRNA library at MOI of 1 and enhanced by spinoculation. Two independent transductions were performed with the same pool, leading to two populations of transduced Jurkat cells. Medium was replaced 24 h later and after 48 h, cells were challenged with HIV-HSA at a MOI of 1, washed 6 h later and cultured in RPMI-10 for 7 days as aforementioned. Medium was replaced every 2 days.

Transduced Jurkat cells challenged with HIV-HSA were negatively selected using a biotinylated anti-HSA antibody (BD Pharmingen, CA, USA) and CELLection Biotin magnetic beads (Invitrogen Dynal, Oslo, Norway), according to the manufacturers' protocol. This iterative procedure was repeated for 3 more rounds. Cells were recovered, cultured in RPMI-10 supplemented with 2 µg/ml of puromycin and grown for 7 days. Procedures of viral infection, negative selection and cell culture with puromycin were performed 3 times. The negatively-selected puromycin resistant-cells were cloned with a ClonaCell™-TCS semi-solid medium (StemCell Technologies, Vancouver, Canada) and grown in 96-well plates with RPMI-10 supplemented with 2 µg/ml of puromycin. The resistant cell clones were expanded and cells were allowed to growth for 2 months.

### Immunoblotting

Intracellular Gag and Vif protein expression was evaluated by western blot. Briefly, cells were washed in ice-cold PBS and lysed in RIPA lysis buffer for 30 min at 4°C. Protein concentration was quantified by Bradford colorimetric assay (BioRad, CA, USA). Equal amounts of protein were analyzed by 12% SDS-PAGE, transferred onto nitrocellulose membranes (GE Healthcare, Buckinghamshire, UK), blotted with anti-p24 primary antibody (AIDS Research and Reference Reagent Program, Division of AIDS, NIAID, NIH, #530, from Dr. Susan Zolla-Pazner), anti-vif primary antibody (AIDS Research and Reference Reagent Program, Division of AIDS, NIAID, NIH, #2221, from Dr. Dana Gabuzda) and anti-GAPDH primary antibody (6C5; Santa Cruz Biotechnology, CA, USA), followed by incubation with HRP-conjugated secondary antibodies (BioRad) and developed using the ECL (GE Healthcare) or Femto (Pierce, IL, USA).

### EGFP-Encoding Lentiviral Particles Production and shRNA Clones Transduction

To produce eGFP-encoding lentiviruses particles, HEK293T cells were co-transfected with pGagPol [15], pRev [15], pFugW [16] and pHEF-VSVG (AIDS Research and Reference Reagent Program, Division of AIDS, NIAID, NIH, from Dr. Lung-Ji Chang) or pSVIIIexe7pA-HxB2 [17] in the proportion of 2∶1∶1∶0.2. After 48 h, lentiviral particles were collected and quantified by p24^CA^ ELISA. shRNA clones were transduced with the eGFP-encoding lentiviral particles at a MOI of 1 and enhanced by spinoculation. After 6 h, cells were washed and medium was replaced. After 48 h post-transduction cells were collected for flow cytometry analysis.

### Flow Cytometry Analysis

Cells transduced with HIV-HSA were harvested at 2, 4 and 7 days and cells transduced with FUGW-VSV or FUGW-GP120 were harvested after 48 h. Afterwards, washing with 1% BSA-PBS, HIV-HSA-transduced cells were stained for HSA surface expression (BD Pharmingen) for 30 min at 4°C. Then, cells were washed and fixed with 0.1% formaldehyde in PBS. Cells transduced with FUGW-VSV or with FUGW-GP120 were washed and fixed with 0.1% formaldehyde in PBS. Labeled untransduced cells were used as negative controls. BD FACS Calibur (BD Bioscience, CA, USA) was used to acquire at least 10,000-gated events from each sample. Data was analyzed using FlowJo software (Tree Star, OR, USA).

### Transient Transfection Assays

HeLa-P4 cells were co-transfected with 300 ng of pLKO.1 shRNA for each target gene and 100 ng of pHIV-1_NL4-3_. Transfections were performed with FuGENE® HD (Roche, IN, USA) according to manufacturers' protocol. After 48 h, viral particles were collected and quantified by p24^CA^ ELISA. Cells were collected to evaluate *β-galactosidase* expression by a colorimetric assay, based on the cleavage of chlorophenolred-β-D-galactopyranoside (CPRG; Roche) as described in [18].

### Assessment of Cell Viability

After 7 days of infection, shRNA clones viability was determined using the Cell Proliferation Reagent WST-1 (Roche) according to manufacturer's instructions.

### Statistical Analysis

Statistical significance was determined using the Paired t-test. Differences were considered statistically significant when p≤0.05. Analyses were performed using the Graphpad Prism 4.0 software (GraphPad Software, CA, USA).

## Results

### shRNA Screening to Isolate HIV-1 Resistant Jurkat T-Cells

**Figure 1:**
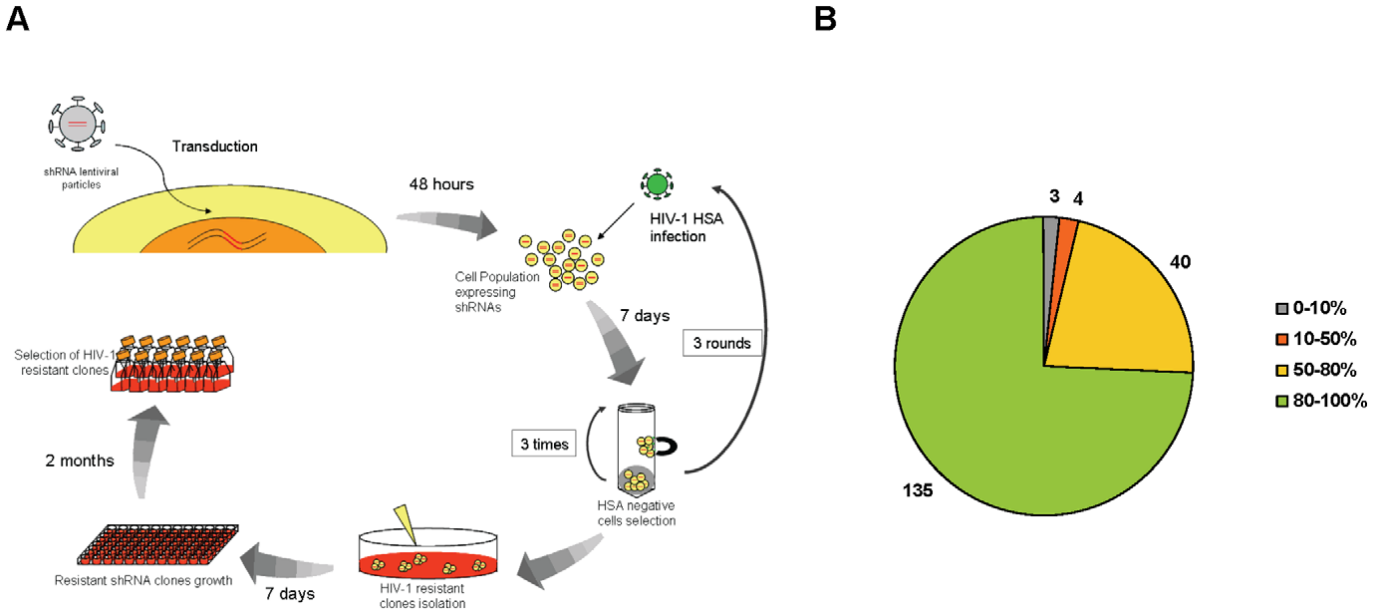
shRNA screen in Jurkat cells. **A**. Schematic representation of the shRNA screen. A pool of shRNA-encoding-lentiviral particles was used to transduce Jurkat cells and after 48 h they were challenged with HIV-HSA. After 7 days of infection, the shRNA transduced cells were negatively selected with magnetic beads conjugated with biotinylated anti-HSA. After 3 rounds of infection/selection, the HIV-1 resistant clones were recovered and isolated. Seven-hundred shRNA Jurkat clones were obtained, expanded and allowed to growth for 2 months to identify cellular proteins essential for HIV-1 replication in Jurkat cells but not essential for the cell viability. We obtained 180 viable shRNA clones. **B**. Resistance of shRNA clones to HIV-1 replication, measured by p24^CA^ expression in the cell culture supernatant after 7 days of infection with HIV-1_NL4-3_ (MOI of 1). Percentage values are relative to Jurkat cells infected with HIV-1_NL4-3_. Values indicated in graph represent the number of clones isolated in each subgroup.

To identify host factors essential for HIV-1 replication we developed a shRNA screen in Jurkat T-cells using a subset of the RNAi consortium (TRC) lentiviral library highly enriched for human kinases and phosphatases This library includes 2855 clones corresponding to 622 human kinase genes and 735 clones corresponding to 180 human phosphatase genes, together with 1693 clones corresponding to other human genes. The list of total genes included in the enriched library will be disclosed upon request. As described in Figure 1A, shRNA encoding lentiviral particles were produced and used as a pool to transduce Jurkat cells at an MOI of 1 (1×10^6^ TU/ml quantified by p24^CA^ ELISA). Experimental conditions were optimized to achieve a highly efficient VSV-G dependent lentiviral transduction of ∼75% (*data not shown*). Lentiviral library transduction was performed twice in 5×10^6^ cells to increase the odds of having all shRNAs transduced in our Jurkat population. Due to high transduction efficiency and the reduced number of individual clones in this library compared to genome-wide representation, we found that the probability of all shRNA clones to be transduced is P(A')  = 0,998. Subsequently, transduced cells were challenged with a replication competent HIV-1 encoding murine heat stable antigen HSA used as a cell surface marker to discriminate between infected and non-infected cells. After 7 days of HIV-1 infection, we selected shRNA-transduced cells HSA-surface-negative and potentially resistant to HIV-1 replication. This procedure was performed in 3 consecutive rounds to assure an enrichment of cells resistant to HIV-1 with the removal of HSA-expressing cells from the system. The remaining HSA-negative cells were cultured in the presence of puromycin to select expressing shRNA Jurkat cells. Following negative and puromycin selections, cells were individually cloned and expanded to evaluate resistance to HIV-1 infection.

We obtained 700 individual shRNA-transduced Jurkat cell clones resistant to HIV-1 replication. To identify kinases and phosphatases essential for HIV-1 replication but innocuous for T-cell viability, individual shRNA clones were expanded and cultured for 2 months in medium supplemented with puromycin. At this period, the number of Jurkat shRNA clones that survived was reduced to 184, which may be due to cytotoxic effects resulting from gene knockdown in cells cultured for 60 days.

To further confirm that viable shRNA clones were resistant to HIV-1 replication, an infection assay was performed for each individual clone. All 184 shRNA clones were infected with HIV-1_NL4-3_ with a MOI of 1. After 7 days of infection, viral replication was measured by p24^CA^ ELISA in supernatant of infected cultures and resistance to HIV-1 replication was determined. As shown in Figure 1B, the majority of shRNA clones were highly resistant to HIV-1 replication. Indeed, when compared to wild-type Jurkat cells, 136 out of 184 shRNA clones exhibited more than 80% reduction in HIV-1 replication, indicating that our original shRNA screen was able to efficiently isolate T-cells clones resistant to HIV-1 replication.

### Identification of the shRNAs Targets

To identify HIV-1 dependent host-factors targeted by the shRNA that were responsible for viral resistance, we selected 30 shRNA Jurkat cell clones with highest resistance to HIV-1 replication and sought to identify their shRNA sequences after genomic DNA extraction and PCR amplification. The shRNA clones analyzed lead to the identification of 14 different gene targets, as described in Table 1. From the 30 sequences we have identified cellular host-factors with only one shRNA gene-specific sequence and genes that were targeted by more than one shRNA.

**Table 1:** Proteins identified in the shRNA screen important for HIV-1 replication.

Gene Symbol	Name	Functional Category	# shRNA clones
PTPN9/PTP-MEG2	Protein tyrosine phosphatase, non-receptor type 9	Member of the protein tyrosine phosphatase (PTP) family	10
PTPRE	PTPRE protein tyrosine phosphatase, receptor type, E	Member of the protein tyrosine phosphatase (PTP) family	1
PRKD1/PKD/PKC-MU	Protein kinase D1	Member of the protein kinase C (PKC) familyCytosolic serine/threonine kinase	2
MAP3K2/MEKK2	Mitogen-activated protein kinase kinase kinase 2	Serine/threonine protein kinaseMember of MEK kinase family	1
MAPK9/JNK2	Mitogen-activated protein kinase 9	Member of the MAP kinase family	1
SGK/SGK1	Serum/glucocorticoid regulated kinase	Serine/threonine protein kinase	1
STK24/MST3	Serine/threonine kinase 24 (STE20 homolog, yeast)	Upstream of the mitogen-activated protein kinase (MAPK) cascade	1
CIB2	Calcium and integrin binding family member 2	Ca^2+^-binding regulatory protein that potentially interacts with DNA-dependent protein kinase catalytic subunit (DNA-PKcs)	1
PPFIA2	Protein tyrosine phosphatase, receptor type, f polypeptide interacting protein (liprin), alpha 2	Member of the LAR protein-tyrosine phosphatase-interacting protein (liprin) family	
PPFIBP1	PTPRF interacting protein, binding protein 1 (liprin beta 1)	Member of the LAR protein-tyrosine phosphatase-interacting protein (liprin) family	1
RAD23B	RAD23 homolog B (*S. cerevisiae*)	Protein involved in the nucleotide excision repair (NER)	1
EZH2	Enhancer of zeste homolog 2	Member of the Polycomb-group	1
WT1	Wilms tumor 1	Transcription factor	3
ELA1/CELA1	Elastase 1, pancreatic	Serine protease	4

As shown in Table 1, amongst the 14 host-proteins identified as essential for HIV-1 replication we found 2 phosphatases, 5 kinases, 1 hypothetical kinase-binding-protein, 2 phosphatase-binding-proteins and 4 other proteins with various functions. By performing biochemical functional analysis (IPA–Ingenuity Systems) the identified proteins were clustered in two major groups: Amino Acid Metabolism, Post-Translational Modification, Small Molecule Biochemistry and Cell Cycle, Cell Signaling, Cellular Growth and Proliferation [(Supporting Information S1, Figure S1)]. The specific molecular and/or cellular functions are represented in Figure S2A.

The identification in this screen of proteins other than kinases or phosphatases is due to the fact that this was an enriched shRNA library that contained other target genes besides kinases and phosphatases. The significant number of non-kinase and non-phosphatase genes in our output compared with its percentage in the initial library could be justified by the strategy of long-term screening. After two months of selection with puromycin and infection with HIV-1, a large number of shRNA clones were not detected. These clones may constitute a mixture of shRNA that induced different levels of cytotoxicity in the cell. During more than 60 days in culture, viable shRNA clones may outgrowth other Jurkat-T-cell clones that showed decreased cytotoxicity over time. Therefore, since kinases and phosphatases are involved in biochemical and cell-cycle pathways essential for proliferation and cell survival, the long-term selection method could have eliminated the less viable clones from the final output. In this study, we have evaluated all resultant genes including non-kinases and non-phosphatases, except for PTPRE that was not studied due to technical reasons.

### Identified Host-Proteins Are Essential for HIV-1 Replication

Next, we wanted to exclude the possibility that integration of shRNA lentiviral vector showed off-target effects. Thus, we recloned 3-to-5 shRNA for each host-protein identified in the previous assay, creating Jurkat cell lines stably expressing the corresponding shRNA (each shRNA sequence is reported in Table S1). The resistance of these shRNA clones to HIV-1 replication was evaluated by replication assays (Figure S3) and the two most efficient shRNA clones were selected. To confirm effectiveness of the shRNA clones selected we performed a quantitative real-time PCR (qPCR), with specific primers (Table S2), assessing mRNA downregulation for each target gene (Figure S4). The real-time assays were performed for all genes in study. In the majority of the shRNA clones a denoting decrease is observed in gene-specific mRNA levels indicating a down-modulation of the genes by the shRNAs. However, for some shRNA clones the mRNA level reduction was not so evident. For ELA1 gene both shRNA clones show lower diminution in mRNA levels (figure S4E), but the replication assays indicate a high reduction in HIV-1 replication. These results may indicate that a slight alteration in ELA1 mRNA levels reflects a considerable alteration in phenotype, as observed in Figure S3E. For PPFIA2-2 and SGK-2 shRNAs, the results showed a minor reduction in mRNA levels, although for PPFIA2-1 and SGK-1 shRNA the knockdown is stronger (Figure S4I and S4M).

The following studies assessing the inhibitory effect on HIV-1 replication were performed with the chosen shRNA clones. Subsequent to clone expansion, shRNA clones were infected with HIV-1_NL4-3_ at a MOI of 1 to determine the extent of viral replication in these cells. After 7 days of infection we determined viral replication by quantifying the amount of virion capsid protein p24^CA^ in cell culture supernatant by ELISA. To assure that the RNAi pathway was activated by the stable expression of shRNAs and did not interfere with HIV-1 replication leading to off-target effects, we used scrambled shRNA (shSCRAM) as control. This is a non-specific shRNA that activates the RNAi pathway, without targeting any human genes.

**Figure 2:**
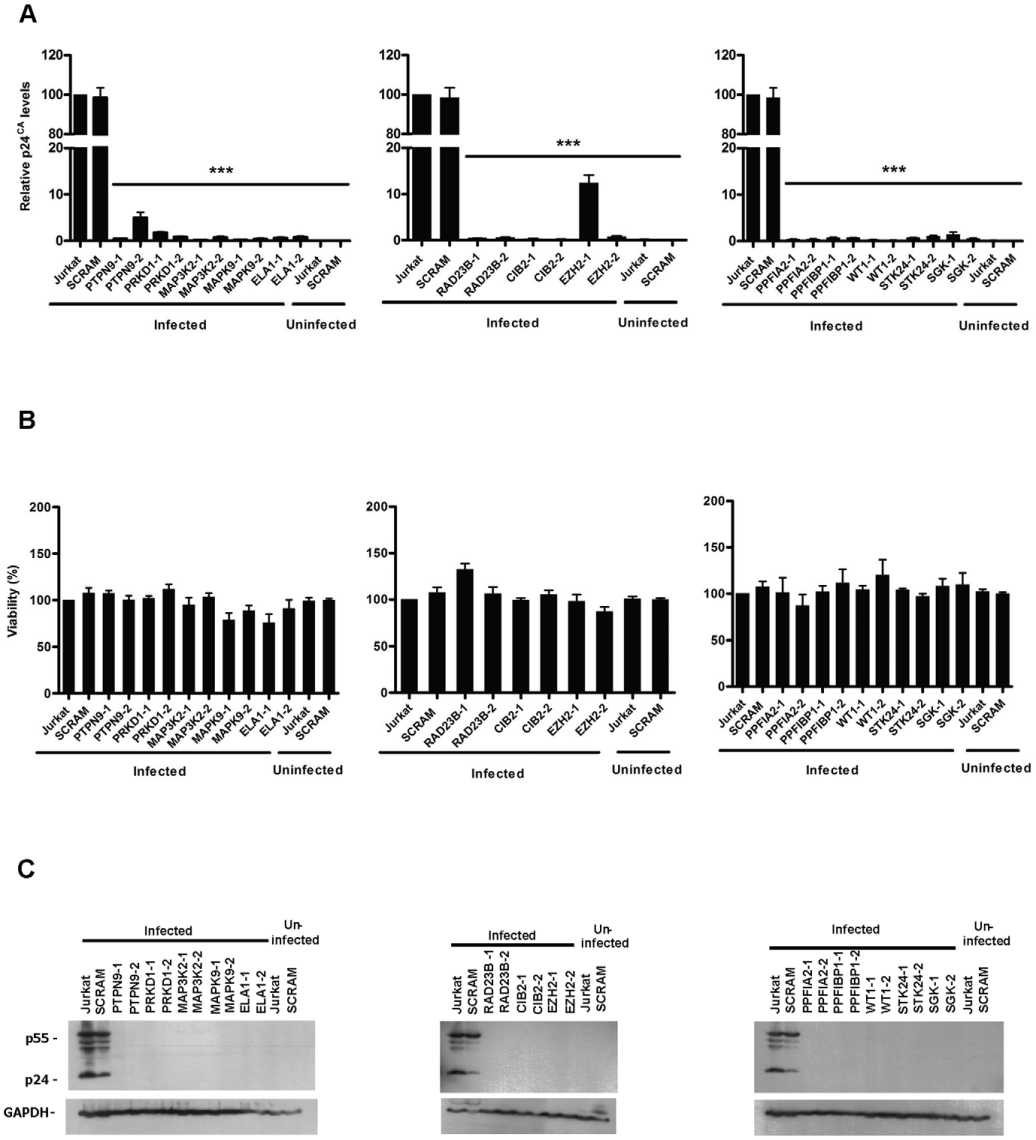
shRNA clones are resistant to HIV-1 replication. **A**. Two different shRNA clones for each target gene were infected with HIV-1_NL4-3_ (MOI of 1) and after 7 days of infection, HIV-1 replication was measured by p24^CA^ levels in the cell culture supernatant. Values are relative to Jurkat cells infected with HIV-1_NL4-3_ and represent the mean ± SEM (*n* = 6). *** corresponds to *P*<0,0001. **B**. Viability of shRNA clones after 7 days of infection with HIV-1_NL4-3_. Values are relative to Jurkat cells infected with HIV-1_NL4-3_ and correspond to mean ± SEM (*n* = 4). **C**. Immunoblotting of intracellular Gag protein in different shRNA clones after 7 days of HIV-1_NL4-3_ infection (MOI of 1). This figure is representative of three independent experiments.

As showed in Figure 2A, HIV-1 replication was strongly inhibited in all shRNA clones tested (over 80% of inhibition), supporting the relevance of these host-proteins during HIV-1 replication. Importantly, inhibition of HIV-1 replication was not due to an outcome of decrease cell viability as all shRNA clones exhibited viability values similar to the control (Figure 2B).

To test if inhibition of HIV-1 replication was a result of a lower viral expression or instead a reduction in viral release, we analyzed the intracellular *gag* gene expression of shRNA T-cell clones by western-blot. As shown in Figure 2C, after 7 days of infection with HIV-1_NL4-3_, neither P55^Gag^ nor p24^CA^ were detected in any of the shRNA Jurkat clones except for shSCRAM (control). These results corroborate the p24^CA^ ELISA data and strengthen the important role of targeted host-proteins in a step(s) prior to virus expression.

### Efficient Knockdown Is of HIV-1 Replication Is Maintained Overtime

**Figure 3:**
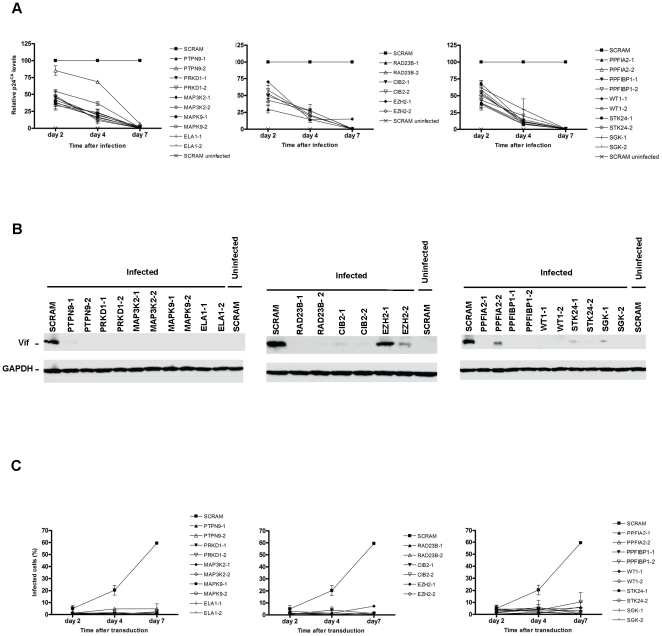
Neutralization of HIV-1 replication by shRNAs is cumulative over time. **A**. Kinetics of HIV-1_NL4-3_ replication in shRNA Jurkat clones during 7 days of infection. shRNA clones were infected with HIV-1_NL4-3_ (MOI of 1) and p24^CA^ antigen was measured at day 2, 4 and 7. Values are relative to control shSCRAM cells infected with HIV-1 (▪) and represent mean ± SEM (*n* = 3). **B**. Immunoblotting of Vif protein in the different shRNA clones after 48 h of HIV-1_NL4-3_ infection (MOI of 1). This figure is representative of three independent experiments. **C**. Flow cytometry analysis of HSA surface expression in shRNA clones infected with HIV-HSA (MOI of 1) during a time course assay of 7 days of infection. Cells were membrane stained with anti-HSA antibody for detection of HIV-1 infection. Percentage of infected cells was analysed by flow cytometry. Values correspond to mean ± SEM (*n* = 2).

To evaluate whether inhibition of HIV-1 replication in the shRNA clones occurred early in viral replication or rather if it would reflect a cumulative effect, we followed HIV-1 infection over time and assessed viral spread in culture. We infected shRNA T-cell clones with HIV-1_NL4-3_ and monitored viral production overtime by measuring the p24^CA^ in cell culture supernatant at day two, four and seven (Figure 3A). As represented in the three panels of Figure 3A, we can observe a pattern of viral replication throughout time for all different shRNA clones. Compared to HIV-1 replication in shSCRAM cells normalized to 100% at all time points, we observed a continuous reduction in the amount of virus in supernatant of shRNA infected clones. p24^CA^ levels were lower at day 2 (25% to 75% depending the shRNA clone) and were constantly reduced until day 7 where nearly no p24^CA^ was detected.

To determine the effect of shRNAs in the expression of a *de novo* viral protein we evaluated by western-blot the expression of Vif after 48h of HIV-1_NL4-3_ infection. As shown in Figure 3B, the expression levels of Vif were barely undetectable compared to control (shSCRAM). The greater reduction of Vif expression (Figure 3B) compared with p24^CA^ levels in Figure 3A can be explained by the detection of residual input p24^CA^ from virus still present at 48 h and not resulting from a *de novo* replication. Therefore, when considered together, these results indicate that inhibition of HIV-1 replication by gene-specific shRNAs is very effective and initiates early in HIV-1 replication. Nevertheless, for EZH2-1 shRNA and in minor importance for EZH2-2 and PPF1A2-2 shRNAs, expression of Vif was higher than for other knockdowns. Taking in consideration the reduction in viral replication observed with these gene-specific shRNA, this fact may reflect a mechanism of HIV-1 inhibition that is subsequent to viral expression.

To get better understanding on the blocking effect of host-protein expression by shRNA in HIV-1 infection and dissemination in culture, we challenged the different shRNA clones with the HIV-HSA reporter virus. This system allows HIV-1 infection to be followed at single-cell level by enumerating HSA^+^ cells by flow cytometry after surface staining. Seven-day time course assay demonstrated that while the percentage of shSCRAM infected cells steadily increased from less than 10% at day 2 to approximately 60% at day 7, the percentage of HSA^+^ of all shRNA clones remained relatively unchanged through time (Figure 3C). In this experiment we also monitored HIV-1-HSA replication by p24^CA^ ELISA and observed a similar pattern in all shRNA clones compared to replication of the parental HIV-1 (Figure S5) indicating that both HIV-1_NL4-3_ and HIV-HSA replication in the shRNA clones were performed in an akin way. To evaluate if the lower percentage of infected cells over time could result from the loss of CD4 expression at the cell surface, we monitored the CD4 positive cells during 7 days of infection with HIV-1_NL4-3_ (Figure S6). We observed a lack of significant decrease of CD4 surface expression in shRNA clones compared with wild-type Jurkat cells or shSCRAM clone.

Despite the observation that shRNAs do not completely knockdown host-proteins gene expression (Figure S4), viral replication was strongly reduced as demonstrated in Figure 3. These results support the hypothesis that an effect of host-proteins on viral replication is highly sensitive to small variations in protein expression which is reflected in an immediate effect of shRNA.

### Knockdown of Host-Proteins Do Not Affect Integration but Rather Affect an Early Step in HIV-1 Replication

**Figure 4:**
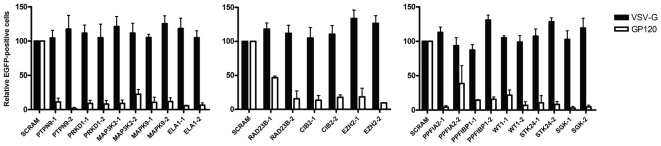
Knockdown of host-proteins do not affect integration but affect an early step in HIV-1 replication. Jurkat shRNA clones were transduced with EGFP-expressing lentiviral particles (FugW-EGFP) pseudptyped with VSV-G and HIV-1 gp120. After 48 h, EGFP expression was measured by flow cytometry. Values are relative to the percentage of shSCRAM EGFP positive cell and represent mean ± SEM (*n* = 3). Black bars indicate values for shRNA clones transduced with a VSV-G-lentivirus and white bars indicate values to shRNA clones transduced with a GP120-lentivirus.

The observation that *gag* products were not detected intracellularly, lead to the hypothesis that the identified host-proteins would be important in an early stage of HIV-1 life cycle, before Gag expression (Figure 2C). To further investigate whether HIV-1 replication cycle was affected before or after viral integration knockdown-Jurkat cells were transduced with an HIV-1-based lentiviral vector, carrying the EGFP transgene, and pseudotyped with a VSV-G envelope. Expression of the fluorescent protein was under the control of human ubiquitin-C promoter to avoid a bias effect that host-proteins might be involved in LTR-driven expression. Analysis of EGFP expression at 48 h post-transduction by flow cytometry showed similar fluorescence results compared to the control shSCRAM indicating that in all Jurkat-knockdown-cells HIV-1 proviral vector was efficiently integrated (Figure 4, black bars). To assess whether by using VSV-G-pseudotyped HIV-1 vectors we were overcoming an entry defect in Jurkat knockdown clones, similar experiments were performed with a gp120-pseudotyped lentiviral vector. Results from transduction experiments showed that all shRNA Jurkat clones express low levels of EGFP compared to Jurkat shSCRAM (Figure 4, white bars). Taken together, these results indicate that these newly identified proteins do not affect viral integration but have a role during HIV-1 entry.

**Figure 5:**
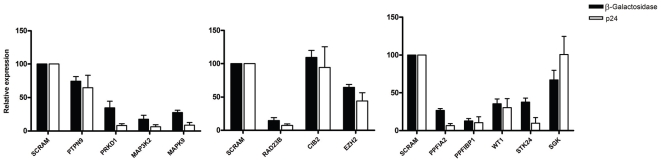
HIV-1 LTR-driven transcription is affected by host-proteins. Transient assays were performed in HeLa-P4 cells co-transfected with pHIV-1_NL4-3_ and the different shRNA plasmids. After 48 h, cells were harvested and LTR transcription was measured by quantification of the β-Galactosidase activity in cell lysates. Cell supernatant was also collected to measure viral production by p24^CA^ ELISA. Black bars indicate values for measurements of β-Galactosidase activity in cell lysates and white bars indicate values for measurements of p24^CA^ in the supernatant. Values are relative to the control shSCRAM and represent mean ± SEM (*n* = 3).

HIV-1 LTR-driven transcription is affected by host-proteins. Since these newly identified host-proteins are cellular transcription factors we sought to assess whether its knockdown could affect Tat transactivation of HIV-1 expression. The effect of knockdown host-proteins was monitored in HeLa-P4 cells containing the *β-galactosidase* gene under the control of the HIV-LTR. HeLa-P4 cells were transiently cotransfected with pHIV-1_NL4-3_ and an individual gene-specific shRNA. The efficiency of LTR-driven expression was determined by β-Galactosidase activity 48 h post-transfection. By examining β-Galactosidase activity (Figure 5, black bars), the relative expression of shRNA against PRKD1, MAP3K2, MAPK9, RAD23B, EZH2, PPFIA2, PPFIBP1 and WT1 exhibited a decrease on LTR-directed transcription compared with control shSCRAM. However, no noteworthy effect could be observed for shRNA against PTPN9, CIB2 and SGK. Concomitantly, levels of p24^CA^ antigen in supernatant were also determined to assess viral production in one cycle of infection (Figure 5, white bars). Our data showed that shRNAs with an effect on HIV-1 transcription were associated with a significant decrease in the p24^CA^ levels. Taken together, our results strongly suggest that the decrease in viral production in this context is a direct consequence of reduced LTR-driven expression when PRKD1, MAP3K2, MAPK9, RAD23B, EZH2, PPFIA2, PPFIBP1 and WT1 are knockdown. Furthermore, for PTPN9, CIB2 and SGK shRNAs, neither β-Galactosidase nor p24^CA^ levels were affected by shRNA expression, suggesting that these proteins do not play an important role in transcription of HIV-1.

## Discussion

Current anti-HIV therapies targeting viral proteins have significant constraints. One strategy to overcome current limitations is to target cellular proteins that are less variable than viral proteins. Kinases and phosphatases are key drug targets and subject of intense scrutiny due to their wide role in cell signalling and other biochemical activities. Hence, the aim of this study was to identify cellular proteins, in particular kinases and phosphatases that would be essential for HIV-1 replication but innocuous for the cell. Consequently, these proteins can potentially be used as antiviral targets. For this purpose, we explored a shRNA library enriched for all human kinases and phosphatases and performed an iterative shRNA screen in Jurkat T-cells. Previous studies have performed RNAi screens at a genome-wide-scale to identify possible host factors that could assist HIV-1 infection [7]–[10]. These genetic screens were all performed with the same goal but the resultant proteins did not overlap in great extent. These results can be justified by the different approaches chosen in each study. In the first three studies using siRNA libraries [7]–[9], where the knockdown is transient, different host cell lines were used, different timings of siRNA treatment were employed, and different readout experiments were performed. In addition, in all studies different criteria were chosen for the bottlenecks after the primary screen which could have accentuated the differences in the final results [19]. The more recent screen [10] used a different approach that is closest to the study described in this manuscript. The work of Kuan-Teh Jeang and co-workers performed a more extensive knockdown screen with a genome-wide shRNA library in Jurkat T-cell line. This last work seems to be most advantageous compared to previous studies [20]. Despite the differences, all screens applied a broad approach with an extensive RNAi library that covers all human genes.

Our work focused on a more restricted strategy using a smaller library, enriched against kinases and phosphatases instead of a generalized genome-wide library. With this strategy not only we ensured that this druggable class of proteins was the major focus of the screening but also we bias it representation in the final results diminishing the probability of off-target proteins. Like Yeung *et al.* we decided to perform a screen with a shRNA library to constitutively knockdown the target genes and used Jurkat cells to better mimic the HIV-1 natural host. The main differences between our study and Yeung *et al.* reside on the use of a restricted shRNA library and a different screen platform. We selected HIV-1 resistant cells with a reduction in viral expression (reduced expression of HSA at cell surface) instead of cell survival after HIV-1 infection. With this strategy we assure that HIV-1 resistant Jurkat cells express shRNA that knockdown cellular proteins directly contributing to productive HIV-1 replication. Instead, selection by cell survival can recover shRNA that may interfere with apoptosis, or other mechanisms that promote cell viability. These differences are expected to complement and improve the goal of discovering novel knockdown targets for HIV-1 that can be used as antiviral targets.

With this work we identified 14 different proteins that strongly assist HIV-1 replication. The assessment of critical steps in HIV-1 life cycle allowed us to conclude that none of these proteins seem to a have a relevant role in HIV-1 proviral integration. Instead, all proteins seem to play an important role before viral integration in an early step of HIV-1, entry and/or uncoating. Moreover, our results demonstrate that PRKD1, MAP3K2, MAPK9, RAD23B, EZH2, PPFIA2, PPFIBP1, WT1 and STK24 have an effect on HIV-1 LTR transcription. On contrary, PTPN9, CIB2 and SGK do not show an important outcome on this viral replication step. Therefore, we can infer that PRKD1, MAP3K2, MAPK9, RAD23B, EZH2, PPFIA2, PPFIBP1, WT1 and STK24 may be important for HIV-1 entry (or uncoating) and transcription. Conversely, PTPN9, CIB2 and SGK are only involved in entry/uncoating. This double functionality could be the result of multifunctional protein acting on different replication steps of HIV-1 replication or instead, can be an indirect effect of these proteins on the HIV-1 LTR promoter and other cellular promoters leading to the inhibition of additional proteins involved HIV-1 entry.

None of the 14 proteins identified in this study have been previously reported as directly involved in HIV-1 replication. Although several kinases and phosphatases have been described to contribute to productive HIV-1 replication, [21]–[23], we have not selected them in this screening. Moreover, even when we introduced a bias for selecting kinases and phosphatases, only 57% of resulting proteins belong to this class, when initially 68% were present in the library. This might have occurred due to cell cytotoxicity during the period of more than two months in culture when these proteins were knocked down by shRNA. The relative weak viability of this knockdown cells could have been outcompeted by other shRNA clones against proteins which function was less cytotoxic for the cell. This explanation is particularly important for kinases and phosphatases since these classes of proteins are crucial in processes of cell death and survival. Therefore, interfering with HIV-1 replication by knockdown kinases/phosphatases function can have deleterious effects on cell viability particularly when important kinases are targeted.

Nevertheless, when we overlap our screen with the previous studies [7]–[10], we observed that two proteins were already present in early screens. PTPN9 was identified by Konig *et al.* [8] and CIB2 was reported by Zhou *et al.* [9]. Although, when these authors used more restricted conditions in subsequent screens, these proteins were not identified. The different selection strategies used between these studies and ours can justify this incongruity. Moreover, when we searched our proteins in the NIAID HIV Protein Interaction Database [24] two other important proteins emerge from our results, MAPK9 and PRKD1. In addition, a recent meta-analysis study where several reports of cellular proteins essential for HIV-1 replication and protein databases were intersected, identifies CIB2 as a potential druggable protein important for viral replication [25]. Importantly, in a core analysis to our set of genes aiming to evaluate their biochemical relationships and possible function in the cell, we observed that the canonical pathways more represented in our screen (Figure S2B and Table S4) are also clearly represented in the previous studies [7]–[10], indicating a possible overlap of signal pathways instead of a direct overlap of genes.

Even though our shRNA screen in Jurkat cells has not selected highly-known HIV-1 helper-factors, the links described above validate the strategy presented in this study as efficient to identify important helper factors that assist HIV-1 replication. As mentioned above, the 14 proteins we have identified have not yet been described as directly involved in HIV-1 replication. Nevertheless, the putative involvement of these proteins into different cellular pathways could lead to a better knowledge of their function during HIV-1 life cycle (Table S3). For example, EZH2 was described to interact with EED, a member of the PcG family, at the EED–EZH2 complex in mammals [26]. In turn, EED was described to interact with HIV-1 matrix, integrase and Nef [27]–[29]. Until this date the EED role in HIV-1 life cycle is not well understood. Data suggests that EED could be involved in cellular function(s) necessary in early steps of HIV-1 life cycle [28] or that EED may function as a negative regulator of HIV-1 assembly and release [30]. Our results seem to indicate that EZH2 acts as a positive factor in HIV-1 entry. Another cellular protein demonstrated to be important for HIV-1 is NDR1. A previous work showed that NDR1 and NDR2 are incorporated into HIV-1 virions and the viral protease cleaves these proteins altering its enzymatic activity in order to favour HIV-1 replication [31]. In addition, NDR1 phosphorylation leads to activation of STK24 [32]. Therefore, it is conceivable that STK24 could also be incorporated in HIV-1 and could have an important role in HIV-1 replication. SGK is transcriptionally activated by the Glucocorticoid receptor (GR) [33], which has been demonstrated to interact with HIV viral protein VPR within a complex integrating VIP-1. A study suggested that the interaction VPR-GR could induce apoptosis since NF-kB inhibition by VPR seemed to be GR-dependent [34]. In addition, a recent work has also demonstrated that VPR-GR interactions increases LTR-mediated transactivation, most likely prior to the presence of Tat [35]. The GR recruitment by VPR into the nucleus could activate SGK that would induce LTR-driven expression. This hypothesis correlates with our results where SGK acts a positive factor for HIV-LTR transcription.

Furthermore, a report using chemical inhibitors indicated that ERK pathway via PRKD1 was dramatically activated by Tat in monocytes [36]. Moreover, PRKD1 together with PI3K has a role in presenting the catalytically active form of CDK9 to the HIV-1 promoter. It was also demonstrated that LTR-activation by PRKD1 is Tat-independent [37], corroborating with our findings.

RAD23B is homologous to RAD23A (HHR23A) and both are described to have the same function in Nucleotide Excision Repair (NER) pathway [38]. RAD23A is important for HIV-1 replication, it interacts physically with VPR, which seems to be critical for cell cycle arrest [39], [40]. Hence, RAD23B could be as important to HIV-1 as RAD23A or have a different function in HIV-1 entry and/or HIV-1 transcription as our results indicate.

Finally, the human leukocyte elastase (HLE), also known as ELA2, has been shown to interact with HIV-1 glycoprotein gp41. HLE is proposed to be a rate-limiting receptor for viral entry potentially by a mechanism involving the phenomenon of receptor co-patching [41]. The structural similarities between ELA1 and ELA2 could lead to the hypothesis that ELA1 could have similar function to ELA2. Indeed, our results identify ELA1 as important for HIV-1.

Regarding MAP3K2 and MAPK9, although they were not shown to be directly involved in HIV-1 replication, many reports have described MAPK pathway to be important for HIV-1 replication in several cell types [42]–[47].

Our study brings new perspectives for HIV-1-host interplay. The exact mechanism and pathway(s) where these proteins are involved is a continuous open question. The indication of possible network(s) (Figure S1) between genes and the putative overlap in some signal pathways, will lead us to future work in the cross-talk between HIV-1 replication and cell signalling. Specifically, and now that a picture is emerging from these studies, more work with primary cells should be implemented to better understand the importance of these genes in primary T-lymphocytes.

In summary, the results presented in this report bring new insights for the complex interplay between HIV-1 and its cellular host leading to a novel perspective for the multifunctional role of the cellular proteins in HIV-1 replication. The identification of new kinases and phosphatases essential for viral replication emphasizes the cell signalling complexity and instigates for further studies involving cellular pathways and HIV-1 replication. Moreover, we show the feasibility to identify host factors that are both essential to the virus and non-essential to the cell. Importantly, these HIV-1 helper-factors being druggable can have a significant impact for new antiviral approaches when traditional strategies fail.

## Supporting Information

Supporting Information S1 Material and Methods
(0.03 MB DOC)

Figure S1 Biochemical relationships between identified proteins. Core analysis was performed with Ingenuity Pathway Analyses (IPA) software (Ingenuity Systems, Inc., CA, USA) to analyse putative relationships between all genes identified in our shRNA screen. The analysis includes only molecules and/or relationships from human specie. Direct (continuous lines) and indirect (dashed lines) relationships are taken into account. Core analysis identified two hypothetical networks between all genes. A. Associated Function Network 1 corresponding to Amino Acid Metabolism, Post-Translational Modification, and Small Molecule Biochemistry: includes ELA1 (CELA1), MAP3K2, MAPK9, PRKD1, PTPN9, PTPRE, RAD23B, SGK1, STK24 and WT1. B. Associated Function Network 2 corresponding to Cell Cycle, Cell Signaling, Cellular Growth and Proliferation and includes CIB2, EZH2, PPFIA2 and PPFIBP1.
(0.67 MB TIF)

Figure S2 Biofunctional analysis of identified genes. A. Molecular and cellular functions of identified genes. Bars indicate de representativeness of genes described in this study. B. Representation of the different canonical pathways wherein the identified genes are present. Bars indicate representativeness in the canonical pathways of genes described in this study. Line represents ratio values between the genes present in each pathway and its representativeness in all canonical pathways. In both analyses threshold value is 0.05. Fisher's Exact Test-P value was performed with IPA software.
(1.79 MB TIF)

Figure S3 Resistance of the different shRNA clones to HIV-1 replication. To evaluate the effect of the different shRNAs to HIV-1 replication, shRNA clones were infected with HIV-1NL4-3 and after 7 days of infection viral replication was measured by p24CA ELISA. A. Evaluation of PTPN9 shRNA clones resistance to HIV-1 replication. shRNA clones PTPN9-B and PTPN9-C were selected to perform the subsequent studies and from than on designated as PTPN9-1 and PTPN9-2, respectively. B. Evaluation of PRKD1 shRNA clones resistance to HIV-1 replication. shRNA clones PRKD1-C and PRKD1-E were selected to perform the subsequent studies and from than on designated as PRKD1-1 and PRKD1-2, respectively. C. Evaluation of MAP3K2 shRNA clones resistance to HIV-1 replication. shRNA clones MAP3K2-A and MAP3K2-D were selected to perform the subsequent studies and from than on designated as MAP3K2-1 and MAP3K2-2, respectively. D. Evaluation of MAPK9 shRNA clones resistance to HIV-1 replication. shRNA clones MAPK9-A and MAPK9-D were selected to perform the subsequent studies and from than on designated as MAPK9-1 and MAPK9-2, respectively. E. Evaluation of ELA1 shRNA clones resistance to HIV-1 replication. shRNA clones ELA1-A and ELA-D were selected to perform the subsequent studies and from than on designated as ELA1-1 and ELA1-2, respectively. F. Evaluation of RAD23B shRNA clones resistance to HIV-1 replication. shRNA clones RAD23B-C and RAD23B-D were selected to perform the subsequent studies and from than on designated as RAD23B-1 and RAD23B-2, respectively. G. Evaluation of CIB2 shRNA clones resistance to HIV-1 replication. shRNA clones CIB2-A and CIB2-B were selected to perform the subsequent studies and from than on designated as CIB2-1 and CIB2-2, respectively. H. Evaluation of EZH2 shRNA clones resistance to HIV-1 replication. shRNA clones EZH2-A and EZH2-C were selected to perform the subsequent studies and from than on designated as EZH2-1 and EZH2-2, respectively. I. Evaluation of PPFIA2 shRNA clones resistance to HIV-1 replication. shRNA clones PPFIA2-A and PPFIA2-C were selected to perform the subsequent studies and from than on designated as PPFIA2-1 and PPFIA2-2, respectively. J. Evaluation of PPFIBP1 shRNA clones resistance to HIV-1 replication. shRNA clones PPFIBP1-C and PPFIBP1-E were selected to perform the subsequently studies and from than on designated as PPFIBP1-1 and PPFIBP1-2, respectively. K. Evaluation of WT1 shRNA clones resistance to HIV-1 replication. shRNA clones WT1-A and WT1-C were selected to perform the subsequent studies and from than on designated as WT1-1 and WT1-2 respectively. L. Evaluation of STK24 shRNA clones resistance to HIV-1 replication. shRNA clones STK24-A and STK24-C were selected to perform the subsequent studies and from than on designated as STK24-1 and STK24-2, respectively. M. Evaluation of SGK shRNA clones resistance to HIV-1 replication. shRNA clones SGK-C and SGK-D were selected to perform the subsequent studies and from than on designated as SGK-1 and SGK-2 respectively. All values are relative to infected Jurkat cells and represent mean ± SEM (n≥3).
(6.82 MB TIF)

Figure S4 mRNA knockdown in individual clones stably expressing shRNA. After recloning shRNA in Jurkat cells, mRNA was extracted from the different shRNA clones and cDNA was purified for posterior quantification by real-time PCR. All graphs represent the two more efficient shRNAs for each gene and mRNA levels are relative to Jurkat cells Values represent mean ± SEM. A. Reduction of mRNA levels in PTPN9 shRNA clones (n = 2). B. Reduction of mRNA levels in PRKD1 shRNA clones (n = 2). und indicate that mRNA levels for these gene were undetectable by qPCR assay. C. Reduction of mRNA levels in MAP3K2 shRNA clones (n = 2). D. Reduction of mRNA levels in MAPK9 shRNA clones (n = 3). E. Reduction of mRNA levels in ELA1 shRNA clones (n = 3). F. Reduction of mRNA levels in RAD23B shRNA clones (n = 3). G. Reduction of mRNA levels in CIB2 shRNA clones (n = 2). H. Reduction of mRNA levels in EZH2 shRNA clones (n = 3). I. Reduction of mRNA levels in PPFIA2 shRNA clones (n = 2). J. Reduction of mRNA levels in PPFIBP1 shRNA clones (n = 3). K. Reduction of mRNA levels in WT1 shRNA clones (n = 3). L. Reduction of mRNA levels in STK24 shRNA clones (n = 2). M. Reduction of mRNA levels in SGK shRNA clones (n = 2).
(6.39 MB TIF)

Figure S5 Monitoring HIV-HSA infection in shRNA Jurkat clones. HIV-1 replication kinetics in shRNA clones during 7 days of infection. shRNA clones were infected with HIV-HSA and p24CA expression was measured at day 2, 4 and 7. Values are relative to control shSCRAM infected cells (▪) and represent mean ± SEM (n = 3). A. Evaluation of HIV-HSA replication in shRNA clones for PTPN9, PRKD1, MAP3K2, MAPK9 and ELA1. B. Evaluation of HIV-HSA replication in shRNA clones for RAD23B, CIB2 and EZH2. C. Evaluation of HIV-HSA replication in shRNA clones for PPFIA2, PPFIBP1, WT1, STK24 and SGK.
(0.81 MB TIF)

Figure S6 CD4 surface expression in Jurkat shRNA clones during HIV-1. Jurkat shRNA clones were infected with HIV-1NL4-3 and CD4 positive cells were measured by flow cytometry at day 0, 2, 4 and 7. Dash lines indicate the values for shSCRAM clone. Values represent mean ± SEM (n = 3).
(5.87 MB TIF)

Table S1 shRNA sequences used for each gene in study. For each gene three to five shRNA sequences were cloned in pLK01 for knockdown of gene expression.
(0.07 MB DOC)

Table S2 Oligonucleotide sequence for target-gene cDNA amplification by qPCR.
(0.04 MB DOC)

Table S3 Genes identified in this screen important for HIV-1 replication. Gene information is described, in particular subcellular localization, molecular class and cellular function.
(0.03 MB PDF)

Table S4 Canonical Pathways involving identified genes. Representation of the different canonical pathways where the genes identified in this study can play a role. The representativeness of each pathway in our study population and the ratio of our genes in the canonical pathway were calculated by IPA-Ingenuity Systems Software.
(0.04 MB XLS)
