# Supplementary material for: Novel HIV-1 Knockdown Targets Identified by an Enriched Kinases/Phosphatases shRNA Library Using a Long-Term Iterative Screen in Jurkat T-Cells
Source: PLoS One. 2010 Feb 17;5(2):e9276. doi: 10.1371/journal.pone.0009276 (PMC2822867; doi:10.1371/journal.pone.0009276)
Supplement: Supporting Information S1 — Material and Methods (0.03 MB DOC) [file pone.0009276.s001.doc]

**SUPPORTING INFORMATION**

**Material and Methods**

**shRNA Sequence Identification**

shRNAs of the HIV-1 resistant clones were PCR amplified from genomic DNA. Approximately 5x10^6^ Jurkat cells were lysed in 500 µl of DNA extraction buffer (10 mM Tris-HCl [pH 8.5], 5 mM EDTA, 0.2 % SDS, 0.2 M NaCl, 100 µg/ml of proteinase K), incubated at 37 °C during 3h with agitation, precipitated with one volume of isopropanol, recovered, washed with ethanol (70%), and dissolved in TE buffer (10 mM Tris-HCl, 0.1 mM EDTA). shRNAs sequences were amplified with the following primers: 5’-GGATGAATACTGCCATTTGTCTCG-3’ and 5’‑AGGCCCGAAGGAATAGAAGA-3’. The resultant PCR products were sequenced using an internal primer: 5´-CAGGGCTGTTAGAGAGATAATTGGA-3´ (MACROGEN, Seoul, Korea). To identify the target gene of each shRNA, computation was performed at National Center for Biotechnology Information (NCBI) using the BLAST network service.

**Development of individual shRNA Jurkat cell clones:** After identifying target genes, we selected 3-5 shRNA constructions for each gene from the TRC and generated Jurkat cells expressing a specific shRNA. Briefly, we produced shRNA lentiviral particles and transduced Jurkat cells with each shRNA as described in Material and Methods. Transduced cells were isolated with ClonaCell™-TCS semi-solid medium (StemCell Technologies) and clones were expanded and maintained in RPMI-10 supplemented with 2µg/ml of puromycin (Sigma, MO, USA). shRNA sequenced are described in Table S1.

**Quantitative Real-Time PCR Analysis**

Total RNA was extracted from shRNA clones using Trizol (Invitrogen, CA, USA) according to the manufacturer’s protocol. cDNA was synthesized using SuperScript^TM^ III First-Strand Synthesis SuperMix for qRT-PCR kit (Invitrogen, CA, USA) according to the manufacturer’s protocol. qPCR was performed on an ABI Prism 7300 PCR system (Applied Biosystems, CA, USA), using the Maxima™ SYBR Green qPCR Master Mix (Fermentas, Ontario, Canada). Primer sequences are described in Table S2. For PRKD1, ELA1 and PPFIA2 genes, qPCR was performed using a Maxima™ Probe qPCR Master Mix (Fermenta) and TaqMan® Gene Expression Assays (Applied Biosystems). The relative amount of target gene mRNA was normalized to GAPDH mRNA. Specificity was verified by melt curve analysis for SYBR Green qPCR .
