# Supplementary figures and images for: Novel HIV-1 Knockdown Targets Identified by an Enriched Kinases/Phosphatases shRNA Library Using a Long-Term Iterative Screen in Jurkat T-Cells
Source: PLoS One. 2010 Feb 17;5(2):e9276. doi: 10.1371/journal.pone.0009276 (PMC2822867; doi:10.1371/journal.pone.0009276)

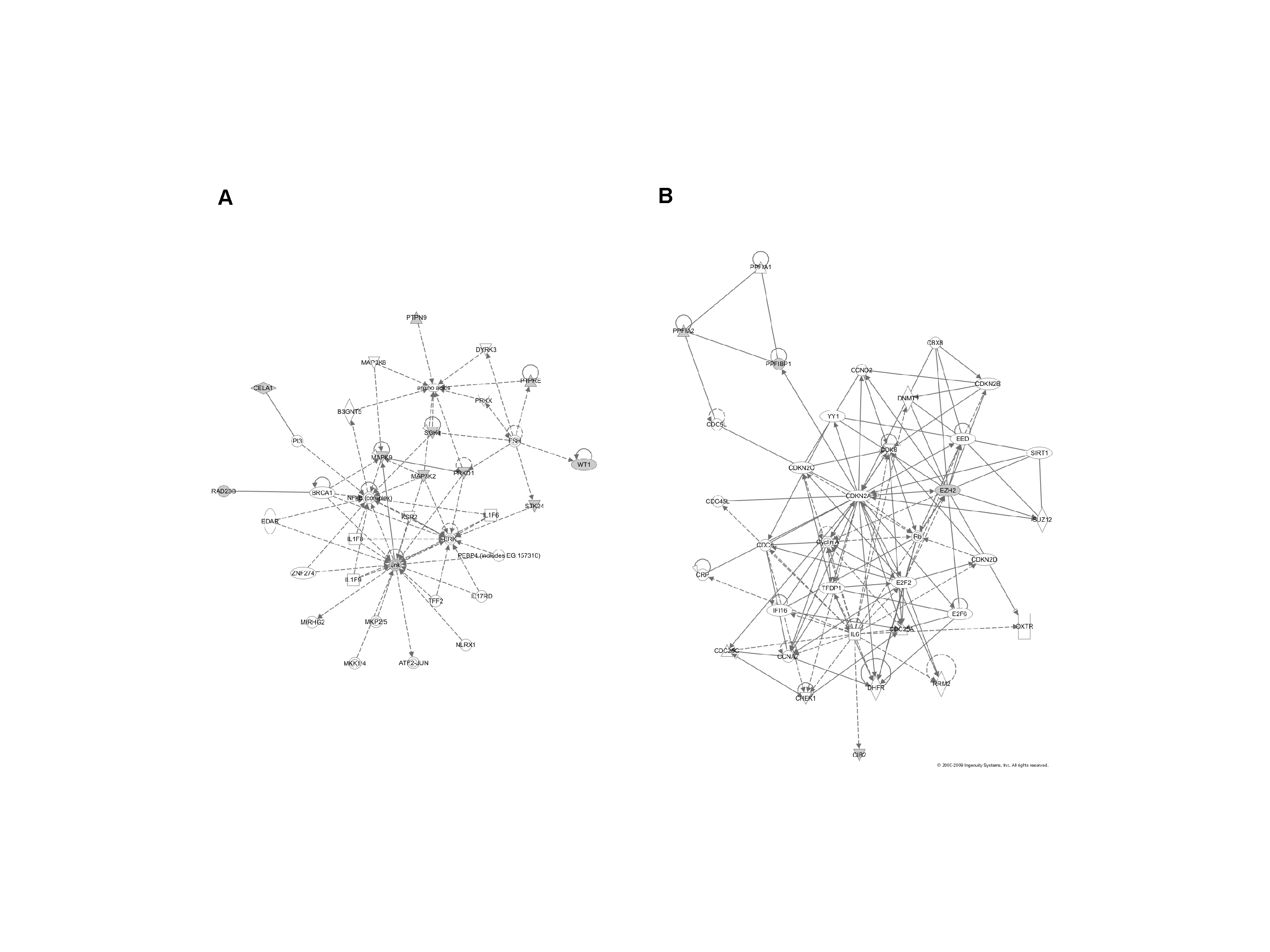

Supplement: Figure S1 — Biochemical relationships between identified proteins. Core analysis was performed with Ingenuity Pathway Analyses (IPA) software (Ingenuity Systems, Inc., CA, USA) to analyse putative relationships between all genes identified in our shRNA screen. The analysis includes only molecules and/or relationships from human specie. Direct (continuous lines) and indirect (dashed lines) relationships are taken into account. Core analysis identified two hypothetical networks between all genes. A. Associated Function Network 1 corresponding to Amino Acid Metabolism, Post-Translational Modification, and Small Molecule Biochemistry: includes ELA1 (CELA1), MAP3K2, MAPK9, PRKD1, PTPN9, PTPRE, RAD23B, SGK1, STK24 and WT1. B. Associated Function Network 2 corresponding to Cell Cycle, Cell Signaling, Cellular Growth and Proliferation and includes CIB2, EZH2, PPFIA2 and PPFIBP1. (0.67 MB TIF) [file pone.0009276.s002.tif]

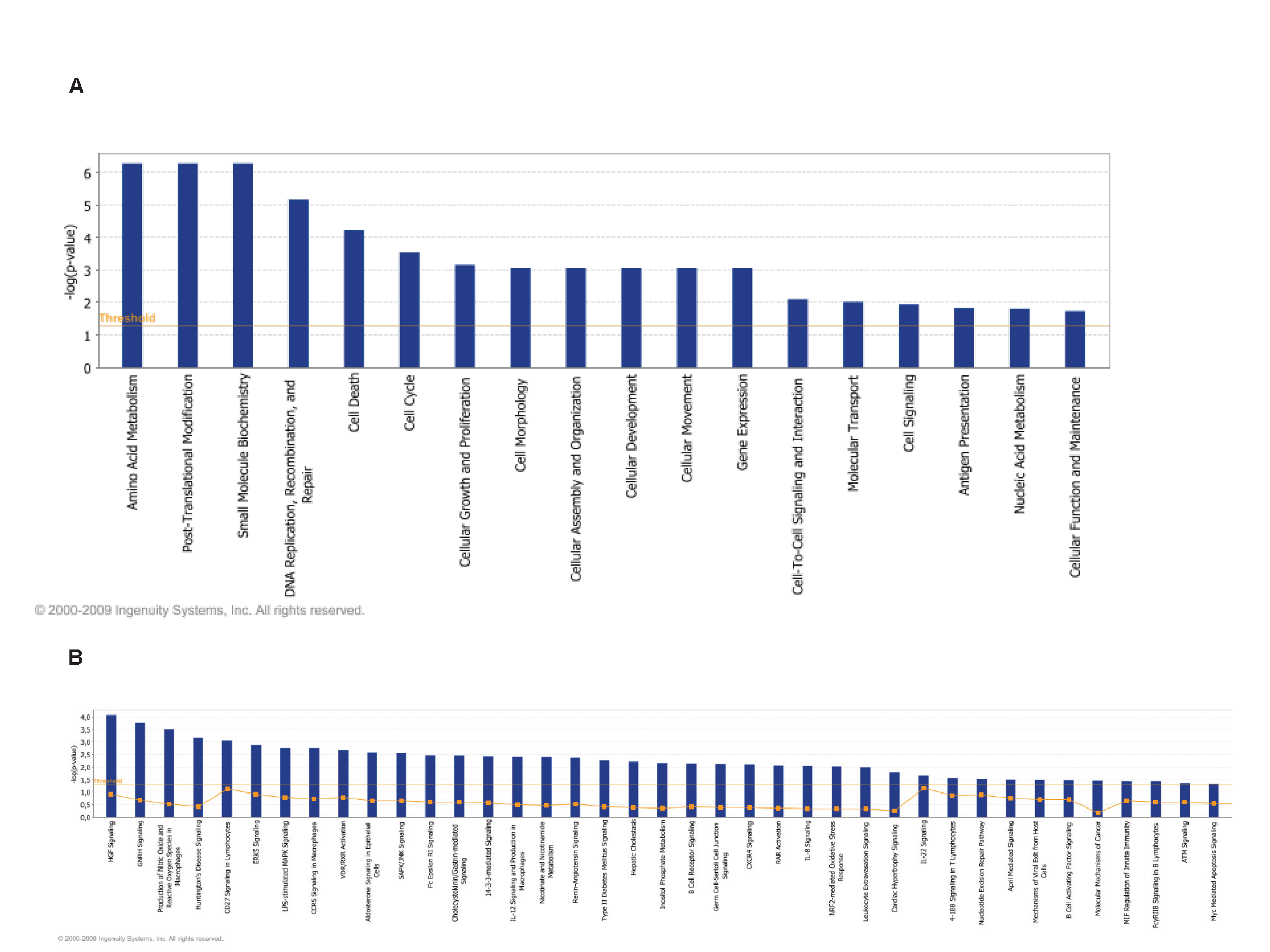

Supplement: Figure S2 — Biofunctional analysis of identified genes. A. Molecular and cellular functions of identified genes. Bars indicate de representativeness of genes described in this study. B. Representation of the different canonical pathways wherein the identified genes are present. Bars indicate representativeness in the canonical pathways of genes described in this study. Line represents ratio values between the genes present in each pathway and its representativeness in all canonical pathways. In both analyses threshold value is 0.05. Fisher's Exact Test-P value was performed with IPA software. (1.79 MB TIF) [file pone.0009276.s003.tif]

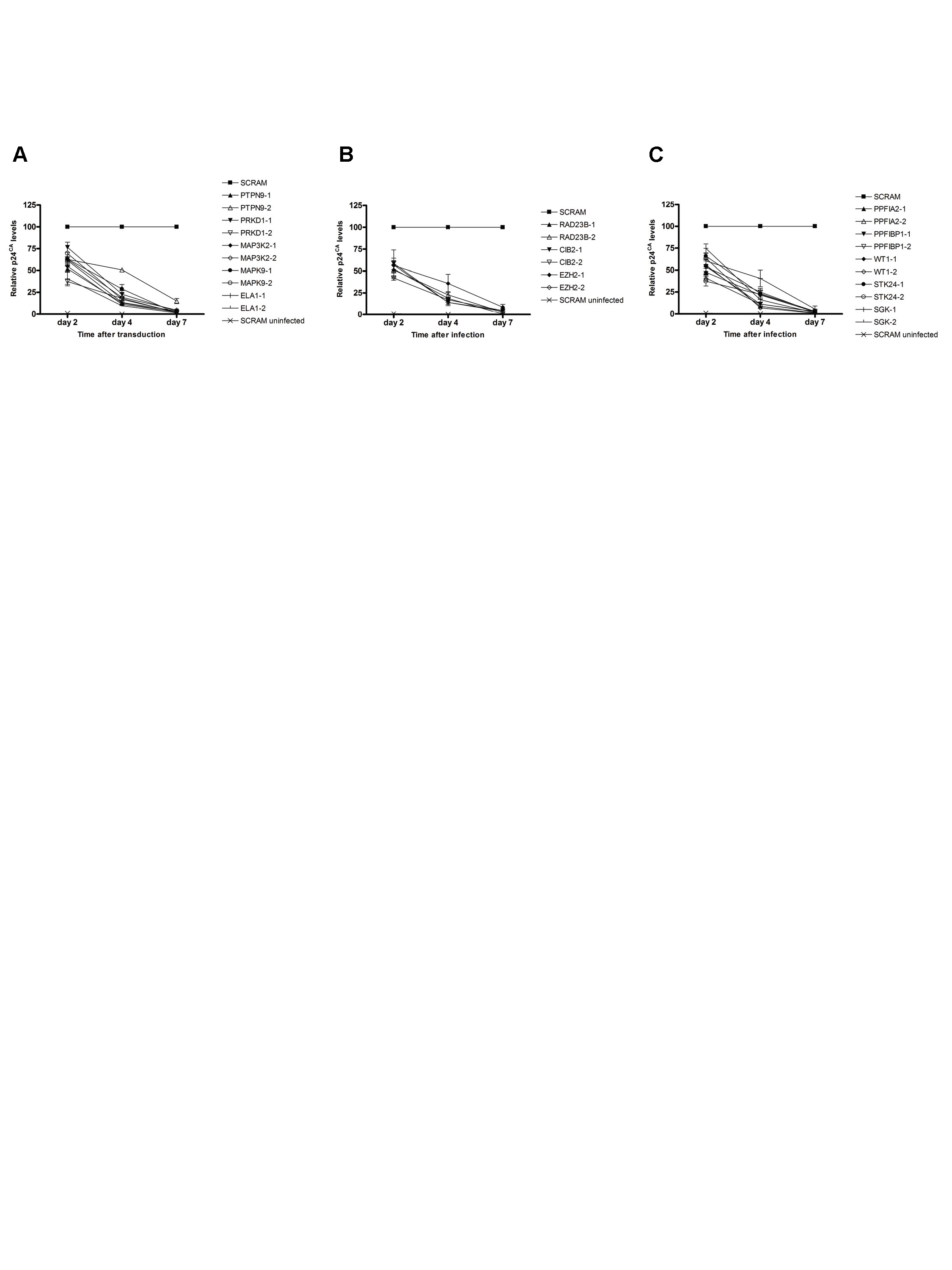

Supplement: Figure S5 — Monitoring HIV-HSA infection in shRNA Jurkat clones. HIV-1 replication kinetics in shRNA clones during 7 days of infection. shRNA clones were infected with HIV-HSA and p24CA expression was measured at day 2, 4 and 7. Values are relative to control shSCRAM infected cells (▪) and represent mean ± SEM (n = 3). A. Evaluation of HIV-HSA replication in shRNA clones for PTPN9, PRKD1, MAP3K2, MAPK9 and ELA1. B. Evaluation of HIV-HSA replication in shRNA clones for RAD23B, CIB2 and EZH2. C. Evaluation of HIV-HSA replication in shRNA clones for PPFIA2, PPFIBP1, WT1, STK24 and SGK. (0.81 MB TIF) [file pone.0009276.s006.tif]
