# Supplementary material for: Novel HIV-1 Knockdown Targets Identified by an Enriched Kinases/Phosphatases shRNA Library Using a Long-Term Iterative Screen in Jurkat T-Cells
Source: PLoS One. 2010 Feb 17;5(2):e9276. doi: 10.1371/journal.pone.0009276 (PMC2822867; doi:10.1371/journal.pone.0009276)
Supplement: Table S1 — shRNA sequences used for each gene in study. For each gene three to five shRNA sequences were cloned in pLK01 for knockdown of gene expression. (0.07 MB DOC) [file pone.0009276.s008.doc]

**Table S1.** shRNA sequences used for each gene in study

| Gene Target | shRNA sequence |
| --- | --- |
| PTPN9 | CCGGGACTATGTTAATGCCAGGCAACTCGAGTTGCCTGGCATTAACATAGTCTTTTT |
|  | CCGGCGAAGGAAGGAAGGCATTGTACTCGAGTACAATGCCTTCCTTCCTTCGTTTTT |
|  | CCGGCCATACTCAGACAGATTACATCTCGAGATGTAATCTGTCTGAGTATGGTTTTT |
|  | CCGGGCTATCAAACTGTGCCTTATTCTCGAGAATAAGGCACAGTTTGATAGCTTTTT |
|  |  |
| PRKD1 | CCGGCCCACGCTCTCTTTGTTCATTCTCGAGAATGAACAAAGAGAGCGTGGGTTTTT |
|  | CCGGCTAAGGAACAAGGGCTACAATCTCGAGATTGTAGCCCTTGTTCCTTAGTTTTT |
|  | CCGGCCATCTCCTATAATCTGTCAACTCGAGTTGACAGATTATAGGAGATGGTTTTT |
|  | CCGGCGGCACTATTGGAGATTGGATCTCGAGATCCAATCTCCAATAGTGCCGTTTTT |
|  | CCGGCCAGAGCACATAACGAAGTTTCTCGAGAAACTTCGTTATGTGCTCTGGTTTTT |
|  |  |
| MAP3K2 | CCGGGCAACGTCAAACTAGGAGATTCTCGAGAATCTCCTAGTTTGACGTTGCTTTTT |
|  | CCGGCCAGATGAATTACACCAGGTTCTCGAGAACCTGGTGTAATTCATCTGGTTTTT |
|  | CCGGCCTTTGGATGGAGAGAGCTATCTCGAGATAGCTCTCTCCATCCAAAGGTTTTT |
|  | CCGGCATATCATCATCAAGAGGTAACTCGAGTTACCTCTTGATGATGATATGTTTTT |
|  |  |
| MAPK9 | CCGGGCTGTCGATGATAGGTTAGAACTCGAGTTCTAACCTATCATCGACAGCTTTTT |
|  | CCGGGATGTGTATTTGGTTATGGAACTCGAGTTCCATAACCAAATACACATCTTTTT |
|  | CCGGCTGTGAGGAATTATGTCGAAACTCGAGTTTCGACATAATTCCTCACAGTTTTT |
|  | CCGGAGGGATTGTTTGTGCTGCATTCTCGAGAATGCAGCACAAACAATCCCTTTTTT |
|  |  |
| ELA1 | CCGGGTACGTGAGTGTGCAGAAGATCTCGAGATCTTCTGCACACTCACGTACTTTTT |
|  | CCGGGAGTGTGCAGAAGATCGTGGTCTCGAGACCACGATCTTCTGCACACTCTTTTT |
|  | CCGGCGTTACCCTCAATAGCTATGTCTCGAGACATAGCTATTGAGGGTAACGTTTTT |
|  | CCGGCTGAAAGACTATTGAGCCATTCTCGAGAATGGCTCAATAGTCTTTCAGTTTTT |
|  | CCGGCTGAAAGACTATTGAGCCATTCTCGAGAATGGCTCAATAGTCTTTCAGTTTTT |
|  |  |
| RAD23B | CCGGCCAGCGTTACTACAGCAGATACTCGAGTATCTGCTGTAGTAACGCTGGTTTTT |
|  | CCGGGTGTACTAGATCCAGAAACTTCTCGAGAAGTTTCTGGATCTAGTACACTTTTT |
|  | CCGGAGAAGCTGGAAGTGGTCATATCTCGAGATATGACCACTTCCAGCTTCTTTTTT |
|  | CCGGCTCCAGCATCAGCGACAGCATCTCGAGATGCTGTCGCTGATGCTGGAGTTTTT |
|  |  |
| CIB2 | CCGGCAACTACCAGGACTGCACCTTCTCGAGAAGGTGCAGTCCTGGTAGTTGTTTTTG |
|  | CCGGCTGACTTCGAGGACATGATTGCTCGAGCAATCATGTCCTCGAAGTCAGTTTTTG |
|  | CCGGCCTCCTTCACAATGTGAAGCTCTCGAGAGCTTCACATTGTGAAGGAGGTTTTTG |
|  |  |
| EZH2 | CCGGCAACACAAGTCATCCCATTAACTCGAGTTAATGGGATGACTTGTGTTGTTTTTG |
|  | CCGGTATGATGGTTAACGGTGATCACTCGAGTGATCACCGTTAACCATCATATTTTTG |
|  | CCGGAAACAGCTGCCTTAGCTTCACTCGAGTGAAGCTAAGGCAGCTGTTTCTTTTTG |
|  |  |
| PPFIA2 | CCGGGCTGAGAAGGATCGAAGACTACTCGAGTAGTCTTCGATCCTTCTCAGCTTTTT |
|  | CCGGCCTACCACAATGATGCTCGAACTCGAGTTCGAGCATCATTGTGGTAGGTTTTT |
|  | CCGGCCTCCATTACTGCCTCTGTTACTCGAGTAACAGAGGCAGTAATGGAGGTTTTT |
|  | CCGGCTAAGAAGACGAGCAGTGAAACTCGAGTTTCACTGCTCGTCTTCTTAGTTTTT |
|  |  |
| PPFIBP1 | CCGGCGGTTAGAGCAGATGGAAGATCTCGAGATCTTCCATCTGCTCTAACCGTTTTT |
|  | CCGGCCAGAGTGTTTCCATTCATATCTCGAGATATGAATGGAAACACTCTGGTTTTT |
|  | CCGGCCTCAATATAAGACCCAGTTTCTCGAGAAACTGGGTCTTATATTGAGGTTTTT |
|  | CCGGGCGTGGATTGTTAGAGATGATCTCGAGATCATCTCTAACAATCCACGCTTTTT |
|  | CCGGGCCAAAGTGAAGCCAAAGAAACTCGAGTTTCTTTGGCTTCACTTTGGCTTTTT |
|  |  |
| WT1 | CCGGCACTCATTCAAGCATGAGGATCTCGAGATCCTCATGCTTGAATGAGTGTTTTTG |
|  | CCGGATGAACTTAGGAGCCACCTTCTCGAGAAGGTGGCTCCTAAGTTCATCTTTTTG |
|  | CCGGTATAAGTACTAGATGCATCACCTCGAGGTGATGCATCTAGTACTTATATTTTTG |
