# Supplementary material for: Novel HIV-1 Knockdown Targets Identified by an Enriched Kinases/Phosphatases shRNA Library Using a Long-Term Iterative Screen in Jurkat T-Cells
Source: PLoS One. 2010 Feb 17;5(2):e9276. doi: 10.1371/journal.pone.0009276 (PMC2822867; doi:10.1371/journal.pone.0009276)
Supplement: Table S2 — Oligonucleotide sequence for target-gene cDNA amplification by qPCR. (0.04 MB DOC) [file pone.0009276.s009.doc]

| **Gene target** | **Primers** |
| --- | --- |
| PTPN9 | agctgaccccggaggag |
|  | gacagccacattccaagaca |
| MAP3K2 | cgtcagattctggagggtgt |
|  | ggccccaaaatctcctagtt |
| MAPK9 | tctttaccagatgctttgtgg |
|  | gtcaaggatcttcagggtgc |
| RAD23B | atgcaggtcaccctgaagac |
|  | gacctgctactggaaaggca |
| CIB2 | accaggactgcaccttcttc |
|  | tctggcatctggatgatgag |
| EZH2 | aaaggatacagacagtgatagg |
|  | cgagaatttgcttcagagga |
| PPFIBP1 | acgtgtggatcactttgctg |
|  | gtcaggggcaaattccaac |
| WT1 | gaaatggacagaagggcaga |
|  | cacatcctgaatgcctctga |
| STK24 | aacaagaaatcacagtgctgagtc |
|  | gcctccaccaagatattcca |
| SGK | tcggactctgcaaggagaac |
|  | atacaagacagctcccaggc |

**Table S2.** Primers sequence for gene target cDNA amplification by qPCR
