# Supplementary material for: Novel HIV-1 Knockdown Targets Identified by an Enriched Kinases/Phosphatases shRNA Library Using a Long-Term Iterative Screen in Jurkat T-Cells
Source: PLoS One. 2010 Feb 17;5(2):e9276. doi: 10.1371/journal.pone.0009276 (PMC2822867; doi:10.1371/journal.pone.0009276)
Supplement: Table S3 — Genes identified in this screen important for HIV-1 replication. Gene information is described, in particular subcellular localization, molecular class and cellular function. (0.03 MB PDF) [file pone.0009276.s010.pdf]

Table 3. Genes identified in this screen as important for HIV-1 replication

Symbol	Synonyms	Name	Gene ID	Subcellular Localization	Molecular class	Molecular function	Biological Importance	References
PTPN9	MEG2 ; PTPMEG2	Protein Tyrosine Phosphatase, non-receptor type 9	NM_002833	Cytoplasm	Tyrosine phosphatase.	Protein tyrosine phosphatase activity	Involved in intracellular traffic of secretory pathways and regulation of vesicular fusion	Huynh, H. et al.2004. *Nat Cell Biol* **6**, 831-839; Saito, K. Et al. 2007. *J Biol Chem* **282**,15170-15178
PTPRE	PTPE; HPTPE; FLJ57799; FLJ58245; DKFZp313F1310; R-PTP-EPSILON	PTPRE protein tyrosine phosphatase, receptor type, E	NM_006504	Plasma membrane/cytoplasm	Receptor tyrosine phosphatase	Receptor signaling protein tyrosine phosphatase activity	Negative regulator of cell proliferation in endothelial cells, of Erk and MAPK pathway in fibroblasts and of Jak-STAT singnalling in M1 myeloid cells	Thompson, L.J. et al. 2001. *Am J Physiol Heart Circ Physiol* **281**, H396-403; Toledano-Katchalski, H.et al.2003. *Mol Cancer Res* **1**, 541-555; Tanuma, N. et al. 2000. *J Biol Chem* **275**, 28216-28221
PRKD1	PKD; PKCM; PRKCM; PKC-MU	Protein kinase D1	NM_002742	Plasma membrane/cytoplasm/Golgi apparatus/Mitochondrion	Serine/threonine kinase. Member of the protein kinase C (PKC) family	Protein serine/threonine kinase activity	Targeted by DAG that regulates PRKD1 intracellular localization. Activated by PKC. Involved in cell growth, gene expression, survival, motility, protein trafficking and lymphocyte biology	Wang, Q.J. et al. 2006 Trends Pharmacol Sci 27, 317-323.
MAP3K2	MEKK2; MEKK2B	Mitogen-activated protein kinase kinase kinase 2	NM_006609	Cytoplasm/nucleus	Serine/threonine kinase. MEK kinase family	Protein serine/threonine kinase activity	MAPK pathway. Leads to ERK1/2, JNK, p38MAPK and ERK5 activation	Chen, Z. et al. 2001. *Chem Rev* **101**, 2449-2476
MAPK9	JNK2; SAPK; p54a; JNK2A; JNK2B; PRKM9; JNK-55; JNK2BETA; p54aSAPK; JNK2ALPHA;	Mitogen-activated protein kinase 9	NM_139069	Cytoplasm/nucleus	Serine/threonine kinase. MAPK family	Protein serine/threonine kinase activity	MAPK cascade. With a controversial role: Studies indicate that is a negative player in cell proliferation; others refute, presenting it as a positive regulator of c-Jun.	Chen, Z. et al. 2001. *Chem Rev* **101**, 2449-2476; Sabapathy, K. et al. 2004. *Cell Cycle* **3**, 1520-1523; 24. Jaeschke, A. et al. *Molecular Cell* **23**, 899–911.
SGK	SGK1	serum/glucocorticoid regulated kinase 1	NM_005627	Cytoplasm/Endoplasmic Reticulum/nucleus	Serine/threonine kinase	Protein serine/threonine kinase activity	Like PKB, SGK is activated by phosphorilation in response to signals that stimulate PI3K, and this is mediated by PKD1 and other unknown kinases. Various proteins were identified as SGK substrates such as GSK3, the transcription factor FOXO3, Raf kinase, the MEKK319 and recently NF-kB20.	Loffing, J. et al. 2006. *Annu Rev Physiol* **68**, 461-490; Tai, D.J. et al. 2009. *J Biol Chem* **284**, 4073-4089.
STK24	MST3; STK3; MST-3; MST3B; STE20	Serine/threonine kinase 24 (STE20 homolog, yeast)	NM_003576	Cytoplasm/nucleus	Serine/threonine kinase	Protein serine/threonine kinase activity	Regulation of cell cycle by phosphorilation and subsequent activation of NDR protein, leading to NDR pathway activation which control cell shape and cell cycle	Stegert, M.R. et al. 2005. *Mol Cell Biol* **25**, 11019-11029
CIB2	KIP2	Calcium and integrin binding family member 2	NM_006383	Cytoplasm/nucleus (?)	Calcium binding protein	Calcium ion binding	Protein with unknown function. Isolog of KIP/CIB gene (DNA PKcs interacting protein)	Seki, N, et al. 1999. Biochim Biophys Acta**1444**, 143-7
PPFIA2	FLJ41378; MGC132572	Protein tyrosine phosphatase, receptor type, f polypeptide interacting protein (liprin), alpha 2	NM_003625	Plasma membrane/Cytoplasm	Anchor protein	Cytoskeletal anchoring activity	Alter PTPRF cellular localization and induces clustering	Serra-Pages, C. et al. 1998. *J Biol Chem* **273**, 15611-20
PPFIBP1	L2; hSGT2; hSgt2p	PTPRF interacting protein, binding protein 1 (liprin beta 1)	NM_003622	Plasma membrane/Cytoplasm	Anchor protein	Cytoskeletal anchoring activity	Role in tumorigenesis	Kriajevska, M. et al. 2002. *J Biol Chem* **277**, 5229-35
RAD23B	P58; HR23B; HHR23B; RAD23B	RAD23 homolog B (S. cerevisiae)	NM_002874	Cytoplasm/nucleus	DNA repair protein	DNA binding	RAD23A homologous. Has a function in DNA repair (NER pathway) and protein stability by ubiquitin-proteosome system regulation.	van Hoffen, A. et al. 2003. *Toxicology* **193**, 79-90; Sweder, K & Madura, K. 2002. *J Biomed Biotechnol* **2**, 94-105
EZH2	ENX1; EZH1; KMT6; ENX-1; MGC9169	Enhancer of zeste homolog 2	NM_004456	Nucleus	Transcription regulatory protein. Member of the Polycomb-group (PcG) family	DNA binding	Involved in maintaining the transcriptional repressive state of human genes by DNA methylation over sucessive cell generations to assure cellular identity	Vire, E. et al. 2006. *Nature* **439**, 871-4.
WT1	GUD; WAGR; WT33; WIT-2	Wilms tumor 1	NM_024424	Nucleus	Transcription factor	Transcription regulator activity	Involved in: regulation of cell proliferation ; regulation of gene expression, epigenetic ; cell differentiation ; hemopoiesis	Morrison, A.A. 2008 *Biochimica et Biophysica Acta* **1785**, 55–62; Yang, L. 2007. *Leukemia* **21**, 868-876
ELA1	CELA1	Elastase 1, pancreatic	NM_001971	Extracellular region/Endoplasmic reticulum/Cytoplasmic vesicle Secretory granule	Serine Protease	Serine-type peptidase activity	Hydrolyses many other protein than Elastase. Seems to activate -kB, AP-1, and NFAT in human myeloid cell.	Hietaranta, A. et al. 2004. *Biochem Biophys Res Commun* **323**, 192-6.
